# Selenium and cancer risk: Wide-angled Mendelian randomization analysis

**DOI:** 10.1002/ijc.33902

**Published:** 2021-12-24

**Authors:** Shuai Yuan, Amy M. Mason, Paul Carter, Mathew Vithayathil, Siddhartha Kar, Stephen Burgess, Susanna C. Larsson

**Affiliations:** 1 Unit of Cardiovascular and Nutritional Epidemiology, Institute of Environmental Medicine, Karolinska Institutet, Stockholm, Sweden; 2 British Heart Foundation Cardiovascular Epidemiology Unit, Department of Public Health and Primary Care, University of Cambridge, Cambridge, UK; 3 National Institute for Health Research Cambridge Biomedical Research Centre, University of Cambridge and Cambridge University Hospitals, Cambridge, UK; 4 Department of Medicine, University of Cambridge, Cambridge, UK; 5 MRC Cancer Unit, University of Cambridge, Cambridge, UK; 6 MRC Integrative Epidemiology Unit, University of Bristol, Bristol, UK; 7 MRC Biostatistics Unit, University of Cambridge, Cambridge, UK; 8 Department of Public Health and Primary Care, University of Cambridge, Cambridge, UK; 9 Unit of Medical Epidemiology, Department of Surgical Sciences, Uppsala University, Uppsala, Sweden

**Keywords:** cancer, kidney cancer, Mendelian randomization, selenium

## Abstract

Evidence on the association between selenium and cancer risk is inconclusive. We conducted a Mendelian randomization study to examine the associations of selenium levels with 22 site-specific cancers and any cancer. Single nucleotide polymorphisms (SNPs) strongly associated with toenail and blood (TAB) and blood selenium levels in mild linkage disequilibrium (r^2^ < .3) were used as instrumental variables. Genetic associations of selenium-associated SNPs with cancer were obtained from the UK Biobank including a total of 59 647 cancer cases and 307 914 controls. Associations with *P* < .1 in UK Biobank were tested for replication in the FinnGen consortium comprising more than 180 000 individuals. The inverse-variance weighted method accounting for linkage disequilibrium was used to estimate the associations. Genetically predicted TAB selenium levels were not associated with the risk of the 22 sitespecific cancers or any cancer (all 22 site-specific cancers). Similarly, we observed no strong association for genetically predicted blood selenium levels. However, genetically predicted blood selenium levels showed suggestive associations with risk of kidney cancer (odds ratio [OR] per one-unit increase in log-transformed levels: 0.83; 95% confidence interval [CI]: 0.67-1.03) and multiple myeloma (OR: 1.40; 95% CI: 1.02-1.93). The same direction of association for kidney cancer but not for multiple myeloma was observed in FinnGen. In the metaanalysis of UK Biobank and FinnGen, the OR of kidney cancer was 0.83 (95% CI: 0.69-1.00). Our study suggests that high selenium status may not prevent cancer development. The associations for kidney cancer and multiple myeloma need to be verified in well-powered studies.

## 1 Background

Selenium, an essential trace mineral, is incorporated into selenoproteins that exert a wide range of effects on human health,^1^ including cancer development.^2^ Both insufficient and excessive dietary intake and circulating levels of selenium can impair health, but there is a lack of consensus concerning the safe range of selenium exposure, especially for cancer risk.^3^ A large amount of studies has been conducted to examine the associations of in vivo selenium (toenail and blood [TAB] selenium) levels and dietary selenium intake with cancer risk.^4^ Most but not all observational studies found that higher circulating levels of or dietary intake of selenium were associated with lower risk of overall cancer and several site-specific cancers, such as breast, colorectal, lung and prostate cancers.^4–7^ Nevertheless, randomized controlled trials have not supported a protective effect of selenium supplementation on risk of overall cancer or common cancers,^4^ but also raised concerns about the excess risk of developing specific neoplasms like high-grade prostate cancer and skin cancer in selenium-supplemented individuals, together with other established adverse effects, such as type 2 diabetes.^8–10^ The conflicting findings from observational studies and randomized controlled trials make the association between selenium and cancer uncertain.

Mendelian randomization (MR) analysis is an epidemiological approach that can strengthen casual inference by using genetic variants as instrumental variables for an exposure.^11^ The approach can minimize residual confounding because the genetic variants are randomly assorted at conception and therefore uncorrelated with important confounders (eg, environmental and self-adopted factors).^11^ In addition, the influence of reverse causation since genetic variants cannot be modified by the development and progression of diseases after fertilization.^11^ Here, we conducted a two-sample MR study to determine the associations of selenium with cancer risk.

## 2 Methods

### 2.1 Genetic instrument selection

Single nucleotide polymorphisms (SNPs) associated with TAB selenium levels at the genome-wide significance level (P < 5 × 10^−8^) were obtained from a genome-wide metaanalysis in 4162 American adults.^12^ The association test was adjusted for age, sex, smoking status and study-specific covariates.^12^ SNPs strongly associated with blood selenium levels (P < 5 × 10^−8^) were selected as complementary instruments from a genome-wide metaanalysis of 2603 Australian twins and their families (with adjustment for age, sex and relatedness) and 2874 British pregnant women.^13^ SNPs were pruned for linkage disequilibrium (*r*^2^ < .3) and the SNP with the lowest *P* value for the genome-wide association was retained. A total of 11 and 22 SNPs were used as instrumental variables for TAB and blood selenium levels, explaining ~4.5% and ~4.0% of phenotypic variance, respectively (Table 1).^14^

### 2.2 Cancer data sources

We extracted data on associations of selenium-associated SNPs with 22 common site-specific cancers and any cancer (all 22 site-specific cancers) from the UK Biobank study. The UK Biobank study is an ongoing cohort comprising data from 500 000 individuals aged between 37 and 73 years at the recruited baseline (2006-2010). Our study was based on 367 561 participants (198 825 women and 168 736 men) followed until 30 June 2020, after removal of individuals with high relatedness (third-degree relatives or closer), low call rate and excess heterozygosity. Incident and prevalent cancer cases were defined by corresponding codes of the International Classification of Diseases (ICD)-9 and ICD-10 with diagnostic information from electronic health records, hospital episodes statistics data, the National Cancer Registry, death certification data and self-reporting validated by nurse interview. Individuals who emigrated from the United Kingdom without medical record information were treated as controls. The association test was adjusted for age at enrollment (continuous), sex and 10 genetic principal components. For cancers possibly associated with selenium in UK Biobank (P < .1), we replicated the association using data from the FinnGen consortium (R5 release data).^15^

### 2.3 Statistical analysis

For selenium-associated SNPs that were unavailable in the outcome datasets, we searched for proxy SNPs at high linkage disequilibrium (r^2^ ≥ .8) with the specified SNP using an online tool (https://ldlink.nci.nih.gov/). The inverse variance weighted method with multiplicative random effects was used to estimate the causal associations of genetically predicted TAB levels and blood selenium levels with 22 site-specific cancers and any cancer. A matrix of genetic correlations among used SNPs was introduced in the MR analysis model.^16^ The estimate from UK Biobank and FinnGen was combined using the fixed-effects metaanalysis method. We performed a sensitivity analysis excluding the SNP associated with height at the genome-wide significance level (rs921943 in *DMGDH* gene region). The I^2^ was used to assess the heterogeneity in each association. Bonferroni correction was used to account for multiple testing, and associations with P-value ≤.002 are described as significant. Associations with *P* value between ≤.10 and >.002 were regarded as suggestive associations requiring replication. In addition, to minimize over-reliance on P-values,^17^ we judged associations by their magnitude and their statistical precision (95% confidence interval [CI]), and the consistency between analyses for genetically predicted TAB and blood selenium levels. All tests were two-sided and performed using the MendelianRandomization^18^ package in the R software (version 4.0.2).

## 3 Results

**Figure 1:**
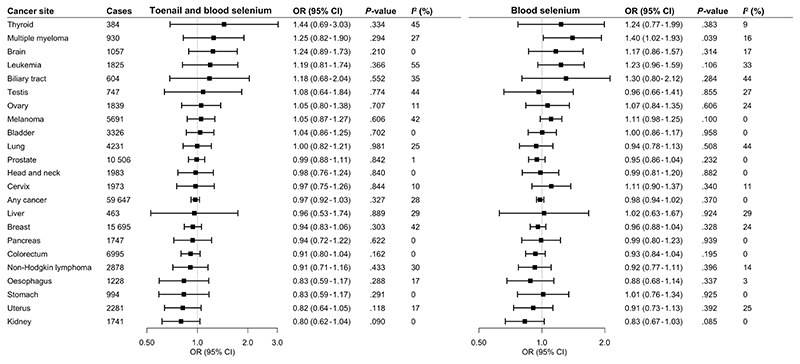
Associations of genetically predicted selenium levels with cancer in UK Biobank. CI, confidence interval; OR, odds ratio. Any cancer includes all 22 site-specific cancers. Estimates represent odds ratios per one unit increase in log-transformed levels

**Figure 2:**
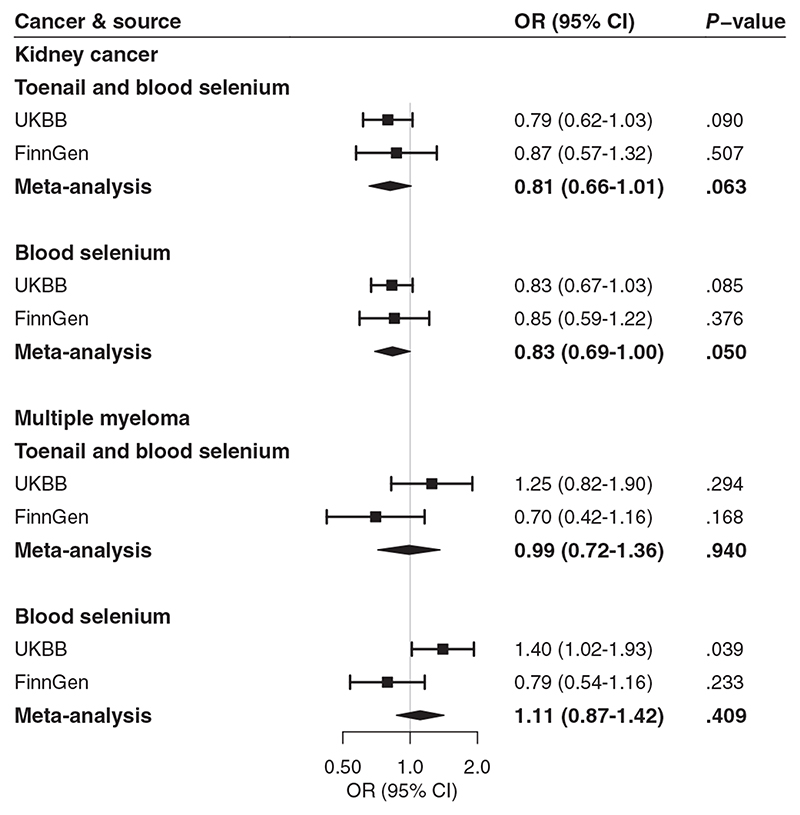
Associations of genetically predicted selenium levels with kidney cancer and multiple myeloma. CI, confidence interval; OR, odds ratio; UKBB, UK Biobank. There were 971 cases and 174 006 controls in the analysis of kidney cancer and 598 cases and 180 756 controls in the analysis of multiple myeloma in FinnGen. Estimates represent odds ratios per one unit increase in log-transformed levels

All selenium-associated SNPs were available in the UK Biobank cancer data. Blood selenium-associated SNPs were all available in the FinnGen consortium data. However, there were three missing SNPs for TAB selenium in the FinnGen consortium data and there were no suitable proxy SNPs. Genetically predicted TAB selenium levels were not associated with the 22 studied site-specific cancers or any cancer (Figure 1) after multiple testing. The odds ratios (ORs) of cancer ranged from 0.80 (95% CI: 0.62-1.04) for kidney cancer to 1.44 (95% CI: 0.69-3.03) for thyroid cancer per one unit increase in log-transformed genetically predicted TAB selenium levels. The associations remained consistent in the complementary analysis using genetic variants associated with blood selenium (Figure 1). Likewise, we observed no association of genetically predicted blood selenium levels with cancers after multiple testing correction. However, higher genetically predicted blood selenium levels were suggestively associated with decreased odds of kidney cancer (OR: 0.83; 95% CI: 0.67-1.03) and increased odds of multiple myeloma (OR: 1.40; 95% CI: 1.02-1.93). The association for kidney cancer but not for multiple myeloma was observed in FinnGen (Figure 2). A suggestive inverse association was observed between genetically predicted blood selenium levels and kidney cancer (OR: 0.83; 95% CI: 0.69-1.00) in the metaanalysis of UK Biobank and FinnGen data. There were possible positive associations of genetically predicted TAB selenium levels with thyroid cancer (OR: 1.44; 95% CI: 0.69-3.03), multiple myeloma (OR: 1.25; 95% CI: 0.82-1.90) and brain cancer (OR: 1.24; 95% CI: 0.89-1.73) (Figure 1). The direction and strength of these associations remained consistent in the analysis for genetically predicted blood selenium levels. In addition, we observed possible positive associations of genetically predicted blood selenium levels with leukemia (OR: 1.23; 95% CI: 0.96-1.59) and biliary tract cancer (OR: 1.30; 95% CI: 0.80-2.12). All associations remained consistent in the sensitivity analysis excluding rs921943 (data not shown).

## 4 Discussion

In our study, we employed MR to assess the potential role of selenium in cancer risk. We found that genetically predicted TAB selenium or blood selenium levels were not associated with cancer risk, except for a suggestive inverse association between genetically predicted selenium levels and kidney cancer. Our study also revealed several possible positive associations for thyroid cancer, multiple myeloma, brain cancer, leukemia and biliary tract cancer. These associations had low precision due to a few cases and therefore warrant future confirmation.

Previous observational studies have generally found inverse associations of selenium intake and circulating selenium levels with risk of any cancer and certain site-specific cancers.^4–7^ However, some subsequent findings disagree with previous results. In a nested case-control study with 743 prostate cancer patients, none of 12 selected selenium pathway genes were associated with prostate cancer risk.^19^ Furthermore, neither toenail selenium levels nor plasma selenoprotein P levels were observed to be associated with prostate cancer risk.^19^ Another nested case-control study including 1186 cancer patients found no association between prediagnostic serum selenium and breast cancer risk.^20^ Likewise, null findings were observed for lung and liver cancer.^21,22^ In a retrospective cohort study including 2065 individuals exposed to high-selenium levels in drinking water and 95 715 unexposed individuals, no significant difference in incidence of any cancer or several common cancers (eg, colorectal, lung, breast and prostate cancers) in the two groups was observed after a 28-year follow-up.^23^

Several randomized controlled trials have been conducted to infer the causality of the associations of selenium supplementation with risk of any cancer, nonmelanoma skin cancer and cancers of the colorectum, lung, breast, bladder and prostate.^4^ None of these trials revealed a possible protective effect of selenium supplementation on preventing incident cancer,^4^ although the overwhelming weight in these analyses was from the Selenium and Vitamin E Cancer Prevention Trial, which included 35 533 men from 427 participating sites in the United States.^4,24^

Previous MR studies have examined the associations of genetically predicted selenium levels with breast,^25^ endometrial,^26^ prostate,^27^ and colorectal^28,29^ cancers. Our study is in line with previous findings on breast and endometrial cancer. However, a possible positive association was observed between genetically predicted selenium levels and advanced prostate cancer in an MR analysis of data from the Prostate Cancer Association Group to Investigate Cancer Associated Alterations in the Genome consortium (OR: 1.21; 95% CI: 0.98-1.49).^27^ We found no evidence of an association between genetically predicted selenium levels and overall prostate cancer in UK Biobank. Higher genetically predicted selenium levels had a suggestive association with a decreased risk of colorectal cancer in an MR study with 58 221 cancer cases and 67 694 controls using SNPs with partial linkage disequilibrium (OR: 0.99; 95% CI: 0.97-1.00).^29^ Our study found a nonsignificant inverse association between genetically predicted selenium levels and colorectal cancer, which might be caused by inadequate power. In addition, we observed a possible inverse association between higher genetically predicted selenium levels and kidney cancer, which is a novel finding that needs confirmation.

A positive association between selenium and multiple myeloma risk has been revealed in several previous studies. A metaanalysis of two trials found a higher risk of hematological malignancies (risk ratio, 1.21; 95% CI: 0.52-2.80) for individuals with selenium administration compared to the controls.^4^ Furthermore, two cohort studies reported a higher risk of multiple myeloma in a population accidentally exposed to unusually high levels of inorganic selenium through drinking water.^23,30^ Our MR study found a suggestive positive association between selenium levels and multiple myeloma in the UK Biobank, which is in line with previous studies. However, we did not replicate this association in the FinnGen consortium. In addition, the wide confidence interval of this association because of few multiple myeloma cases indicates the statistical uncertainty of this association, which needs future confirmation in a well-powered study. We also observed several associations for thyroid cancer, brain cancer, leukemia as well as biliary tract cancer. Although the strength of these associations was comparable to that for multiple myeloma in the UK Biobank, the precision of these associations was low (as indicated by broad CIs) due to few cases and therefore more study is needed to verify these findings.

There are several strengths of our study. The major one is the MR design, which can minimize biases from residual confounding and reverse causation. In addition, MR analysis used unmodifiable genetic variants as instrumental variables to mimic the life-time exposure to high selenium levels, which can detect the long-term effect of selenium on cancer as well as overcome the limitation of low compliance to intervention in randomized controlled trials. We systematically assessed the associations for 22 common site-specific cancers and any cancer and mapped the effect of selenium on different cancers.

Limitations need to be considered when interpreting our findings. Even though we used SNPs that explain relatively large variance in TAB and blood selenium levels, we might still have overlooked weak associations for cancers with a small number of cases. For analysis in the UK Biobank, the bias introduced by outcome misclassification might influence our results given no medical record for individuals who lost to follow-up before they developed cancer, like individuals emigrated from the United Kingdom. In addition, the population in the UK Biobank is relatively young. We might have overlooked certain associations, especially for cancers occurring late in life. Sensitivity analyses to evaluate potential horizontal pleiotropy could not be conducted with partially correlated SNPs. Some SNPs were associated with height.^14^ However, a potential pleiotropic effect from height is not likely to have had a major impact on our results for two reasons. First, the positive association between genetically predicted selenium levels and height was much smaller compared to that on cancer risk. In addition, genetically predicted height is positively associated with some cancers only (the strongest magnitude of association has been observed for biliary tract, thyroid, ovarian, kidney and breast cancers) and the associations are modest.^31^ Second, the associations of genetically predicted selenium levels and cancer risk remained directionally consistent in the analysis excluding the pleiotropic SNP. We cannot rule out that the possible positive associations of genetically predicted selenium levels and biliary tract and thyroid cancer is to some extent driven by height. We also could not rule out the possibility that our findings might have been influenced by other unknown pleiotropic effects. Some studies have revealed that the association between selenium and cancer is nonlinear and that the inverse association is only observed at blood selenium levels up to 170 ng/mL.^7^ Nonetheless, a nutrition survey in US people indicated that a trivial proportion of the population had serum levels of selenium >170 ng/mL,^32^ which indicates that our findings based on a linear model would be robust. Selenium exists in several different forms, both organic and inorganic, which are implied in several pathways and have different properties and the effects of selenium on human health differ regarding the dose and forms.^33^ In this MR study, we had no data to perform analysis on different forms of selenium. The best biomarker for selenium status has not yet been identified. Data on reliability of TAB and blood selenium content are controversial and the levels of TAB and blood selenium do not reflect inorganic selenium sources. In addition, as noted previously, there were no shared loci between toenail selenium and blood selenium.^12^

In conclusion, our study found limited evidence in support of inverse associations of genetically predicted TAB and blood selenium levels with cancer risk, which suggests that high selenium status may not prevent the cancer development in the population. The suggestive inverse association between selenium and kidney cancer warrants more study with a large sample size. In addition, the observed possible positive associations for multiple myeloma, thyroid cancer, brain cancer, leukemia and biliary tract cancer also need verification.

## Figures and Tables

**Table 1:** Genetic instruments for selenium

SNP	Chr	Pos	Gene	EA	NEA	EAF	Beta	SE	*P*
*Blood and toenail selenium*									
rs672413	5	78278229	*ARSB*	A	G	0.32	0.116	0.015	5.21E − 14
rs558133	5	78425188	*BHMT*	C	A	0.31	0.102	0.016	5.60E − 11
rs567754	5	78416416	*BHMT*	C	T	0.66	0.138	0.015	8.38E − 20
rs10944	5	78385845	*BHMT2*	T	G	0.49	0.181	0.014	1.13E − 36
rs11951068	5	78304314	*DMGDH*	A	G	0.07	0.189	0.028	1.86E − 11
rs3797535	5	78300397	*DMGDH*	T	C	0.08	0.210	0.026	2.05E − 15
rs705415	5	78291960	*DMGDH*	C	T	0.86	0.141	0.023	4.64E − 10
rs921943	5	78316476	*DMGDH*	T	C	0.29	0.207	0.016	1.90E − 39
rs6859667	5	78745042	*HOMER1*	C	T	0.04	0.254	0.037	4.40E − 12
rs1789953	21	44482936	*CBSL*	T	C	0.14	0.114	0.021	3.40E − 08
rs234709	21	44486964	*CBSL*	C	T	0.55	0.084	0.014	5.23E − 09
rs6586282	21	44478497	*CBSL*	C	T	0.83	0.113	0.019	3.96E − 09
*Blood selenium*									
rs672413	5	78278229	*ARSB*	A	G	0.32	0.117	0.033	1.68E − 08
rs163124	5	78283003	*ARSB*	G	T	0.28	0.148	0.034	5.16E − 12
rs163132	5	78285921	*ARSB*	C	T	0.23	0.168	0.035	7.32E − 14
rs7700970	5	78411324	*BHMT*	T	C	0.32	0.212	0.037	1.72E − 18
rs10514151	5	78303487	*DMGDH*	T	C	0.06	0.209	0.059	1.42E − 08
rs16876394	5	78346769	*DMGDH*	C	T	0.10	0.305	0.048	3.32E − 22
rs16876498	5	78402594	*DMGDH*	C	T	0.10	0.308	0.048	1.23E − 22
rs17823744	5	78344976	*DMGDH*	G	A	0.13	0.285	0.045	8.93E − 22
rs1915706	5	78436211	*DMGDH*	C	T	0.62	0.161	0.031	3.04E − 15
rs2445887	5	78310044	*DMHDH*	A	G	0.46	0.161	0.031	7.63E − 16
rs248380	5	78331741	*DMGDH*	T	C	0.51	0.206	0.029	1.84E − 27
rs3797535	5	78300397	*DMGDH*	T	C	0.10	0.213	0.057	2.42E − 09
rs478651	5	78290682	*DMGDH*	T	C	0.48	0.162	0.035	3.53E − 13
rs586199	5	78397980	*DMGDH*	G	A	0.50	0.201	0.029	2.37E − 26
rs705415	5	78291960	*DMGDH*	C	T	0.88	0.232	0.059	4.56E − 10
rs7710824	5	78297271	*DMHDH*	A	C	0.28	0.161	0.035	3.86E − 13
rs8180502	5	78477017	*DMGDH*	G	A	0.70	0.121	0.035	4.70E − 08
rs921943	5	78316476	*DMGDH*	T	C	0.30	0.246	0.034	9.40E − 28
rs9293761	5	78290215	*DMGDH*	G	A	0.56	0.186	0.032	1.24E − 18
rs949644	5	78442351	*DMGDH*	A	G	0.67	0.167	0.031	2.03E − 16
rs9293769	5	78629346	*JMY*	T	C	0.60	0.123	0.032	1.75E − 09
rs10514159	5	78596044	*JMY*	C	T	0.62	0.135	0.031	7.48E − 12

Abbreviations: Chr, chromosome; EA, effect allele; EAF, effect allele frequency; NEA, noneffect allele; Pos, position based on GRCh37/hg19; SNP, single nucleotide polymorphism.

## Data Availability

This work has been conducted using the UK Biobank Resource. The UK Biobank is an open access resource and bona fide researchers can apply to use the UK Biobank dataset by registering and applying at http://ukbiobank.ac.uk/register-apply/. Further information is available from the corresponding author upon request. Analyses of UK Biobank data were performed under application 29 202. The FinnGen consortium data are publicly available at https://finngen.gitbook.io/documentation/.

## What’s new?

Selenium is a trace mineral important to human health. Too much or too little selenium can both be harmful, but in regard to cancer risk, observational studies and randomized controlled trials have produced conflicting results. Here, the authors conducted a Mendelian randomization study using single nucleotide polymorphisms (SNPs) strongly linked to selenium levels. Genetically predicted selenium levels were not associated with risk of cancer overall, or of any of the 22 site-specific cancers studied. They did find a suggestive inverse relationship between selenium levels and kidney cancer.

## Abbreviations

CI: confidence interval
ICD: International Classification of Diseases
MR: Mendelian randomization
OR: odds ratio
SNPs: single nucleotide polymorphisms
TAB: toenail and blood.

## References

[1] Rayman MP (2012). Selenium and human health. Lancet.

[2] Rayman MP (2005). Selenium in cancer prevention: a review of the evidence and mechanism of action. Proc Nutr Soc.

[3] Vinceti M, Filippini T, Wise LA (2018). Environmental selenium and human health: an update. Curr Environ Health Rep.

[4] Vinceti M, Filippini T, Del Giovane C (2018). Selenium for preventing cancer. Cochrane Database Syst Rev.

[5] Kuria A, Fang X, Li M (2020). Does dietary intake of selenium protect against cancer? A systematic review and meta-analysis of population-based prospective studies. Crit Rev Food Sci Nutr.

[6] Cai X, Wang C, Yu W (2016). Selenium exposure and cancer risk: an updated meta-analysis and meta-regression. Sci Rep.

[7] Hurst R, Hooper L, Norat T (2012). Selenium and prostate cancer: systematic review and meta-analysis. Am J Clin Nutr.

[8] Albanes D, Till C, Klein EA (2014). Plasma tocopherols and risk of prostate cancer in the Selenium and Vitamin E Cancer Prevention Trial (SELECT). Cancer Prev Res (Phila).

[9] Jablonska E, Vinceti M (2015). Selenium and human health: witnessing a Copernican revolution?. J Environ Sci Health C Environ Carcinog Ecotoxicol Rev.

[10] Vinceti MA-O, Filippini TA-O, Rothman KJ (2018). Selenium exposure and the risk of type 2 diabetes: a systematic review and meta-analysis. Eur J Epidemiol.

[11] Burgess S, Thompson SG (2015). Mendelian Randomization: Methods for Using Genetic Variants in Causal Estimation.

[12] Cornelis MC, Fornage M, Foy M (2015). Genome-wide association study of selenium concentrations. Hum Mol Genet.

[13] Evans DM, Zhu G, Dy V (2013). Genome-wide association study identifies loci affecting blood copper, selenium and zinc. Hum Mol Genet.

[14] Rath AA, Lam HS, Schooling CM (2021). Effects of selenium on coronary artery disease, type 2 diabetes and their risk factors: a Mendelian randomization study. Eur J Clin Nutr.

[15] The FinnGen consortium (2021). R5 results of genome-wide association analyses in FinnGen consortium.

[16] Burgess S, Dudbridge F, Thompson SG (2016). Combining information on multiple instrumental variables in Mendelian randomization: comparison of allele score and summarized data methods. Stat Med.

[17] Amrhein V, Fau-Greenland S, Greenland S, Fau-McShane B, McShane B (2019). Scientists rise up against statistical significance. Nature.

[18] Yavorska OO, Burgess S (2017). MendelianRandomization: an R package for performing Mendelian randomization analyses using summarized data. Int J Epidemiol.

[19] Outzen M, Tjønneland A, Hughes DJ (2021). Toenail selenium, plasma selenoprotein P and risk of advanced prostate cancer: a nested casecontrol study. Int J Cancer.

[20] Sandsveden M, Manjer J (2017). Selenium and breast cancer risk: a prospective nested case-control study on serum selenium levels, smoking habits and overweight. Int J Cancer.

[21] Bai Y, Wang G, Fu W (2019). Circulating essential metals and lung cancer: risk assessment and potential molecular effects. Environ Int.

[22] Ma X, Yang Y, Li HL (2017). Dietary trace element intake and liver cancer risk: results from two population-based cohorts in China. Int J Cancer.

[23] Vinceti M, Vicentini M, Wise LA (2018). Cancer incidence following long-term consumption of drinking water with high inorganic selenium content. Sci Total Environ.

[24] Lippman SM, Klein EA, Goodman PJ (2009). Effect of selenium and vitamin E on risk of prostate cancer and other cancers: the Selenium and Vitamin E Cancer Prevention Trial (SELECT). JAMA.

[25] Papadimitriou N, Dimou N, Gill D (2021). Genetically predicted circulating concentrations of micronutrients and risk of breast cancer: a Mendelian randomization study. Int J Cancer.

[26] Kho PF, Glubb DM, Thompson DJ, Spurdle AB, O’Mara TA (2019). Assessing the role of selenium in endometrial cancer risk: a Mendelian randomization study. Front Oncol.

[27] Yarmolinsky J, Bonilla C, Haycock PC (2018). Circulating selenium and prostate cancer risk: a Mendelian randomization analysis. J Natl Cancer Inst.

[28] Cornish AJ, Law PJ, Timofeeva M (2020). Modifiable pathways for colorectal cancer: a mendelian randomisation analysis. Lancet Gastroenterol Hepatol.

[29] Tsilidis KK, Papadimitriou N, Dimou N (2021). Genetically predicted circulating concentrations of micronutrients and risk of colorectal cancer among individuals of European descent: a Mendelian randomization study. Am J Clin Nutr.

[30] Vinceti M, Ballotari P, Steinmaus C (2016). Long-term mortality patterns in a residential cohort exposed to inorganic selenium in drinking water. Environ Res.

[31] Vithayathil M, Carter P, Kar S, Mason AM, Burgess S, Larsson SC (2021). Body size and composition and risk of site-specific cancers in the UKbiobank and large international consortia: a mendelian randomisation study. PLoS Med.

[32] Vogt TM, Ziegler RG, Patterson BH, Graubard BI (2007). Racial differences in serum selenium concentration: analysis of US population data from the third National Health and nutrition examination survey. Am J Epidemiol.

[33] Weekley CM, Harris HH (2013). Which form is that? The importance of selenium speciation and metabolism in the prevention and treatment of disease. Chem Soc Rev.

